# Can we do for other essential medicines what we are doing for the COVID-19 vaccine?

**DOI:** 10.1136/bmjgh-2021-005158

**Published:** 2021-02-19

**Authors:** Adrianna Murphy, Lilian Mbau, Martin McKee, Kara Hanson, Els Torreele

**Affiliations:** 1 Centre for Global Chronic Conditions, Department of Health Services Research and Policy, London School of Hygiene and Tropical Medicine, London, UK; 2 Kenya Cardiac Society, Nairobi, Kenya; 3 Department of Global Health and Population, London School of Hygiene and Tropical Medicine, London, UK; 4 Institute for Innovation and Public Purpose, The Bartlett Faculty of the Built Environment, University College London, London, UK

**Keywords:** COVID-19, vaccines, health policy, health systems, cardiovascular disease

**Summary box**

- The resources that have been mobilised to accelerate the development and distribution of the COVID-19 vaccine are unprecedented.
- We ask what could be achieved if similar resources were dedicated to improving access to medicines and other health technologies for conditions that affect many more people.
- In 2017, an estimated two billion people did not have access to essential medicines, including for diseases with well-established treatment options like cardiovascular disease and diabetes.
- The COVID-19 vaccine experience has shown us the scale and pace of resource mobilisation and innovation that we are capable of – this must now be applied to ensuring equitable access not only to the COVID-19 vaccine but to all essential medicines and health technologies.

By the beginning of 2021 the world welcomed encouraging news on several candidate vaccines for COVID-19. Pfizer-BioNTech, Moderna, Oxford/AstraZeneca and the Chinese BBIBP-CorV vaccines are already rolled out in some high-income countries, and other vaccines may follow. While in low-income and middle-income countries (LMICs) access is lagging, there are several vaccines with significant potential for fighting the pandemic in those LMICs that need them. For instance, Coronavac, developed by China’s Sinovac Biotech, has been approved in China and is undergoing phase III trials in several LMICs, including Brazil, Indonesia, Bangladesh and the Philippines. By early December, Brazil and Indonesia had received over a million doses of the vaccine. The Sputnik V vaccine has been approved in Russia. The Oxford/AstraZeneca vaccine is being rolled out in India. The advantage of these last three vaccines is that they can be transported, stored and delivered using the standard 2°C–8°C cold chain infrastructure (the Pfizer vaccine needs to be kept at −70°C and Moderna at −20°C). Based on currently available information, the Oxford/AstraZeneca vaccine is also likely to cost less than competitor vaccines, partly because of its different design, but also because AstraZeneca has committed to selling it at cost during the pandemic and in perpetuity to LMICs.

The speed of the response to COVID-19 has been made possible by massive public investments aimed at supporting innovation. Since February 2020, the public sector has invested €93 billion in COVID-19 vaccines and therapeutics.^1^ Roughly a third of this investment has come from the USA, 24% from the European Union and 13% from Japan and South Korea. Some governments, especially Britain and the European Union, have also made significant contributions to COVAX, a global initiative to support delivery of affordable COVID-19 vaccines to LMICs that aims to provide two billion doses by the end of 2021. COVAX is led by the WHO and coordinated by the WHO, Gavi, the Vaccine Alliance, and the Coalition for Epidemic Preparedness Innovations with initial funding from the Bill and Melinda Gates Foundation. Much work remains to purchase adequate amounts of COVID-19 vaccines and to distribute them equitably in LMICs,^2^ especially as richer countries buy up initial vaccine supplies, potentially undermining the ability of COVAX to deliver, and many middle-income countries seek to purchase their own supplies, with plans to start vaccinating early 2021.^3^ Nevertheless, the scale and pace of the global mobilisation of resources thus far—including financial investment, political will and advocacy—are remarkable and unprecedented.

Witnessing this remarkable achievement compels us to reimagine what is possible for other global health challenges. What if similar resources were mobilised for other essential medicines and vaccines, including for conditions that affect many more people than COVID-19? Or what if these resources were invested in strengthening health systems and access to health technologies overall in LMICs, integrating COVID-19 vaccination (where vaccination for COVID-19 is warranted) with programmes to vaccinate against other diseases, or to detect, diagnose and treat other common conditions?^4^ How many more lives could be saved or dramatically improved? In 2017, an estimated two billion people did not have access to essential medicines.^5^ These included medicines for neglected tropical diseases, where investment in research and development (R&D) for effective treatments is well below what is needed, as well as for diseases with well-established treatment options such as HIV/AIDS, tuberculosis and malaria, which are often simply unavailable or unaffordable to those who need them. And while coverage of essential vaccinations in low-income countries has increased significantly in the last decade, coverage of newer vaccines like the human papillomavirus vaccine, which protects against the leading cause of cervical cancer, remains very low (7%).^6^ COVID-19 has likely further exacerbated these problems—already scarce resources have been diverted away from these health issues and to COVID-19, medicine supply chains have been disrupted, and millions of patients and their families are at risk of losing their livelihoods, pushing them into poverty.

The problem of access to medicines is also increasingly relevant for non-communicable diseases (NCDs), which now make up 7 of the 10 leading causes of death worldwide.^7^ Among NCDs, cardiovascular disease (CVD) is the leading cause of death, killing almost 18 million people each year. Contrary to the perception of CVD as a ‘disease of the rich’, four out of five CVD deaths occur in LMICs and half of these are among people under 70 years of age, many in the prime of their productive years.^8^ Despite the massive health and economic burden caused by CVD, essential medicines long used for primary and secondary prevention, including blood pressure-lowering drugs and statins, are unavailable or unaffordable to a large proportion of people in some LMICs.^9^ The same is true for fixed-dose combination therapies for hypertension, which combine two to three blood pressure-lowering medications into one pill, improve treatment adherence, lower healthcare costs and reduce major CVD events, even though they are listed on the WHO Essential Medicines List.^10^ Non-vitamin K antagonist oral anticoagulants (NOACs) are a proven safe and effective treatment for non-valvular atrial fibrillation, reducing stroke, intracranial haemorrhage and mortality. Unlike vitamin K antagonist oral anticoagulants that are more frequently used in LMICs, NOACs pose a lower risk of bleeding, are less likely to interact with other drugs or foodstuffs and do not require regular monitoring, all of which make them well suited to resource-poor health systems in LMICs.^11^ In 2019 NOACs were added to the WHO Essential Medicines List, but they remain unavailable or unaffordable in those countries that could benefit from them the most.^11 12^ Insulin, an essential treatment for diabetes, was discovered nearly 100 years ago and its patent sold for only $1 to the University of Toronto. Today, one out of every two people in LMICs who need insulin cannot access it (although lack of access is not limited to LMICs).^13^

Of course, access to essential medicines depends on supportive national policies. Countries can facilitate access by including these medicines in national essential medicines lists, in clinical treatment guidelines, among those drugs reimbursed or subsidised by national health insurance, and by ensuring that distribution systems deliver consistent supplies to drug dispensaries, pharmacies and health facilities. But actions taken at the international level to support R&D, manufacturing, procurement and supply are also crucial. Voluntary licensing, through mechanisms such as the Medicines Patent Pool (MPP), can allow generic companies in LMICs to produce and sell essential medicines still under patent for a fraction of the cost of branded versions. For example, the MPP has estimated that in Sub-Saharan Africa and in low-income and lower-middle income countries in other regions, licensing agreements for NOACs still under patent protection could avert 10 000–31 000 cases of stroke or systemic embolism, or 94 000–281 000 venous thromboembolism events over a period of 4 years.^12^ When voluntary licensing cannot be obtained, countries must be encouraged to use existing legal flexibilities in international intellectual property laws to overcome patent barriers that stand in the way of public health.^14^ Where drugs are long off patent, including the components of fixed-dose combination therapies for hypertension, other strategies are required. We have seen that inclusion of these medicines in the WHO Essential Medicines List alone is not sufficient to support access, so we need more research to understand systemic barriers to access to medicines.^10^ This research should be done in collaboration with communities, using methods that enable co-creation of interventions at the local health system level.^15^ Importantly, it is necessary to place these measures within a comprehensive global strategy, with advocacy at all levels and incorporation of these drugs in international guidelines. This will require much greater commitment by global actors to work together—including multilateral and national health organisations, governments and advocacy groups, pharmaceutical companies and researchers—to identify and address barriers to affordable access to essential medicines.

The priority given to COVID-19 vaccines and other COVID-19 health technologies is warranted. Normal life has been upended by the pandemic. Lives have been lost and livelihoods destroyed, children have missed school, domestic violence has increased, and the mental health of many has been negatively affected. People have suffered critical illness and died alone, separated from their loved ones. Yet this scale of loss and economic devastation was familiar to many people in different parts of the world long before COVID-19, where households have been impoverished by the high cost of healthcare including essential medicines, and patients have had to forgo treatment altogether and risk death due to unavailability or prohibitive prices.^16^ And the pandemic has only made the situation worse. Efforts by the global health community to forewarn inequity in distribution of the COVID-19 vaccine to LMICs and commitments to prevent this inequity are laudable, but for many in these countries there is nothing new about poor access to essential medicines. For NCDs specifically, the United Nations committed in 2014 and again in 2018 to ensure access for all to affordable essential medicines for NCDs. This commitment was reiterated in the WHO target to achieve 80% availability of affordable, safe and efficacious NCD medications and in the Sustainable Development Goals. We are far from realising these intentions, but what the COVID-19 vaccine experience has shown is that it is possible for the international community to try harder when it decides to do so.
